# Control of stem cell differentiation by using extrinsic photobiomodulation in conjunction with cell adhesion pattern

**DOI:** 10.1038/s41598-022-05888-3

**Published:** 2022-02-02

**Authors:** Saitong Muneekaew, Meng-Jiy Wang, Szu-yuan Chen

**Affiliations:** 1 Department of Chemical Engineering, National Taiwan University of Science and Technology, Taipei City, 106 Taiwan; 2 Institute of Atomic and Molecular Sciences, Academia Sinica, Taipei City, 106 Taiwan; 3 Department of Physics, National Central University, Taoyuan City, 320 Taiwan

**Keywords:** Stem-cell biotechnology, Tissue engineering

## Abstract

The induction and direction of stem cell differentiation into needed cell phenotypes is the central pillar of tissue engineering for repairing damaged tissues or organs. Conventionally, a special recipe of chemical factors is formulated to achieve this purpose for each specific target cell type. In this work, it is demonstrated that the combination of extrinsic photobiomodulation and collagen-covered microislands could be used to induce differentiation of Wharton’s jelly mesenchymal stem cells (WJ-MSCs) with the differentiation direction dictated by the specific island topography without use of chemical factors. Both neurogenic differentiation and adipogenic differentiation could be attained with a rate surpassing that using chemical factors. Application of this method to other cell types is possible by utilizing microislands with a pattern tailored particularly for each specific cell type, rendering it a versatile modality for initiating and guiding stem cell differentiation.

## Introduction

In the past decades, regenerative medicine/tissue engineering has become an increasingly important research field, driven by the motivation to replace tissues or organs damaged by a disease or trauma. In particular, implantation of functioning tissues or organs generated in vitro from the patient’s own cells provides a manageable implant source that does not rely on the availability of immunologically compatible donors. The construction of a functioning tissue/organ could be done by 3D bioprinting or steering the self-organization of cells^1–5^, and the ability to initiate and direct differentiation of stem cells or reprogramming of somatic cells is an essential technique for these technologies.

The conventional way to drive stem cell differentiation into a target cell type is by using a set of chemical factors supplemented in a basal maintenance medium. For example, basic fibroblast growth factor (bFGF) is usually needed not only for stem cell proliferation but also for supporting stem cell differentiation^6^. In addition, 3-isobutyl-1-methylxanthine (IBMX) is adopted to promote neurogenic differentiation through elevating intracellular cyclic adenosine monophosphate (cAMP) level which subsequently activates protein kinase A (PKA)^7^. Furthermore, all-trans retinoic acid (ATRA) has been shown to be an effective factor that can up-regulate proteins characteristic of neurogenic differentiation^8,9^. Recently, it has become generally recognized that physical cues rendered by the nanosctruture, micropattern, and mechanical properties of the cell adhesion substrate can be used to enhance stem cell differentiation in a favored direction^10–12^. For instance, it has been shown that cell adhesion substrate with a fiber-like surface structure such as nanofiber and nanoscale ridge/groove could promote the commitment of human mesenchymal stem cells^13^, human embryonic stem cells^14^, human induced pluripotent stem cells^15^, and human decidua parietalis placental stem cells^16^ to the neural lineage. In addition, it has been known that stem cells adhering and conforming to microislands displays an enhancement toward different differentiation outcomes dictated by the shape and size of the microislands^17–19^. For example, it was found that smaller cell adhesion area favors adipogenic differentiation of mesenchymal stem cells while a larger cell adhesion area favors osteogenic differention^18^. It is also found that cell adhesion morphology of a high aspect ratio facilitates neurogenic differentiation^20,21^, whereas an aspect ratio of 1 favors adipogenic differentiation and an aspect ratio of 2 favors osteogenic differentiation^17^. Moreover, such a morphological guiding cue seems also applicable for lineage reprogramming of adult somatic cells^20^.

On the other hand, photobiomodulation, also referred to as low-level light therapy, has been recognized for its efficacy in speeding up wound healing and boosting cell metabolic activities. It is achieved by irradiating cells with light of wavelengths matching the absorption bands of cytochrome c oxidase in mitochondria to activate intracellular signaling pathways via second messengers such as Ca^2+^^22^, NO^23^, and reactive oxygen species (ROS)^24^. It has been reported that photobiomodulation can facilitate the osteogenic differentiation and neurogenic differentiation of stem cells induced by chemical factors^25–30^. Recently, it was demonstrated that adipogenic differentiation of murine bone marrow-derived mesenchymal stem cells could be induced by loading a photosensitizer into the cells and irradiating the cells with light of matched wavelength^31^. This method, referred to as extrinsic photobiomodulation (EPM) hereafter, is basically the same as the photodynamic therapy (PDT) used to eradicate cancer cells or bacteria^32–36^, but with a light dose low enough to avoid killing the cells through apoptosis or necrosis. This renders a dramatically enhanced version of photobiomodulation for attaining a stronger and more reliable modulatory effect on stem cell differentiation, since the amount and location of the photoreceptors for initiating the critical intracellular signaling can be controlled instead of relying on the cells’ own expression.

Based on the above observations, we hypothesized that EPM could be used to induce stem cell differentiation while control of cell adhesion morphology could be used to determine the direction of differentiation, so that the two in combination might be able to replace the chemical factors needed for driving stem cell differentiation into each specific cell phenotype. In this work, to investigate the potential of such a modality, Wharton’s jelly mesenchymal stem cells (WJ-MSCs) were used as the model stem cell for its abilities of self-renewal and differentiation to various cell lineages such as osteocytes^37^, adipocytes^38^, chondrocytes^39^, neural cells^40^, and smooth muscle-like cells^41^. The application of EPM was achieved by using verteporfin as the photosensitizer, which targeted endoplasmic reticulum, and light irradiation with a laser of 690 nm wavelength. Cell adhesion substrates with microislands of various areas and aspect ratios were fabricated by a photolithographic method to produce arrays of poly(ethylene glycol) diacrylate (PEGDA) microislands over 3-(trimethoxysilyl)propyl methacrylate (TMA)-modified glass surface followed by selective collagen deposition onto the microislands. The results show that both the neurogenic differentiation and adipogenic differentiation of WJ-MSCs can be attained with a rate higher than that using chemical factors when the EPM and collagen microislands are applied together. This verifies the hypothesis and reveals the great potential of this modality for application in tissue engineering.

## Results

### Fabrication of collagen microislands

**Figure 1:**
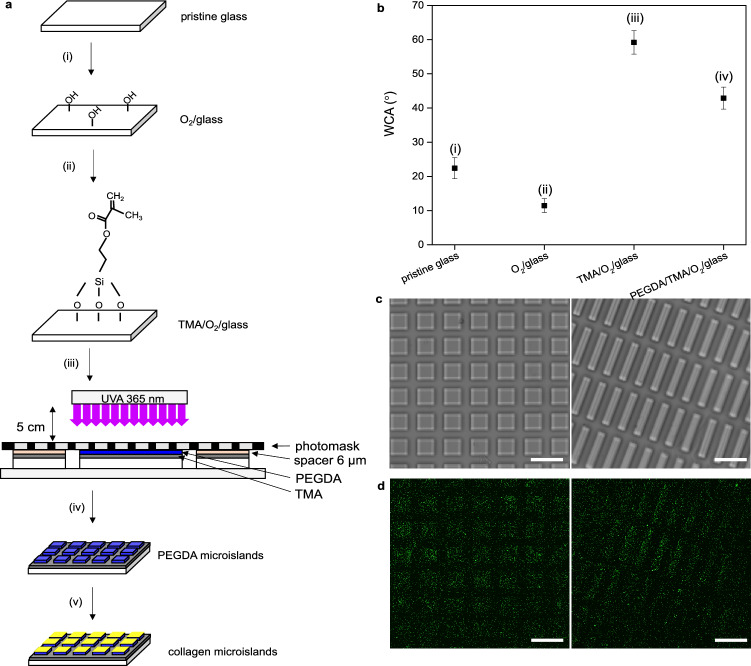
Fabrication of collagen microislands. (**a**) Schematic illustration of the process flow to prepare collagen microislands: (i) the prinstine glass was treated with oxygen plasma for 1 min; (ii) the sample was immersed in TMA solution for 1 h; (iii) the sample was immersed in PEGDA-HOMPP solution and then exposed to UVA (365 nm) through the photomask for 25 s; (iv) microislands was developed by using deionized water to remove uncross-linked PEGDA; (v) collagen was deposited on the microislands by immersing the sample in the collagen solution for 2 h at 37 °C. (**b**) Water contact angle of the surface of the glass after each step of surface treatment: (i) before any treatment (pristine glass); (ii) after oxygen plasma treatment (O_2_/glass); (iii) after TMA treatment (TMA/O_2_/glass); (iv) after PEGDA immersion and non-patterned UVA curing (PEGDA/TMA/O_2_/glass). Data is expressed by mean ± SD (N = 3). (**c**) Phase-contrast microscopy images of 50 μm × 50 μm square collagen microislands and 120 μm × 20 μm rectangular collagen microislands. Scale bar: 100 μm. (**d**) Confocal microscopy images of collagen distribution based on collagen immunostaining for the samples in (**c**).

Collagen microislands were prepared by a series of surface modifications and photolithographic patterning (Fig. 1a). The success of each surface modification step in the fabrication process of collagen microislands and non-patterned collagen film was confirmed by water contact angle (WCA) measurements (Fig. 1b). Oxygen plasma treatment resulted in a decrease of WCA from 22.4 ± 3.1° to 11.5 ± 2.3°, which could be ascribed to the formation of hydroxyl groups on the surface of the glass. After TMA treatment the WCA increased to 59.2 ± 3.4°, indicating successful silanization with TMA. Lastly, the decrease of WCA to 42.9 ± 3.2° revealed the successful deposition of PEGDA on TMA/O_2_/glass via photo-initiated cross-linking of PEGDA and its binding to TMA. In this work, an array of PEGDA microislands of various dimensions was fabricated by using the portion of a photomask with the corresponding pattern during UVA curing, while a non-patterned PEGDA film was obtained by the same method but with a blank photomask. This was then followed by deposition of collagen on PEGDA surface in order to enhance cell adhesion. The PEGDA microislands were evidenced by the phase-contrast images of square islands and rectangular islands fabricated with photomasks of 50 μm × 50 μm pattern size and 120 μm × 20 μm pattern size, respectively (Fig. 1c). With size measurement using ImageJ software, the resultant square microislands exhibited dimensions of 51.2 ± 1.9 μm × 52.1 ± 2.3 μm, while the obtained rectangular microislands displayed dimensions of 120.3 ± 1.1 μm × 20.8 ± 1.5 μm, both very close to the pattern sizes on the photomask, revealing the high definition of this contact exposure method. The spatial distribution of collagen deposited was resolved by using collagen immunostaining and confocal microscopy. It was found that collagen adsorbed only on PEGDA microislands but not TMA (gaps between microislands) (Fig. 1d), resulting in production of collagen microislands at the last step. Note that the fluorescence signal appearing on the gaps between microislands was from the instrumental background, not due to collagen adsorption, because the same signal intensity was obtained in samples without collagen immunostaining. In addition, a collagen coating time of 2 h was necessary to obtain uniform and saturated collagen immunostaining fluorescence intensity on microislands across the entire substrate (Supplementary Fig. S1).

**Figure 2:**
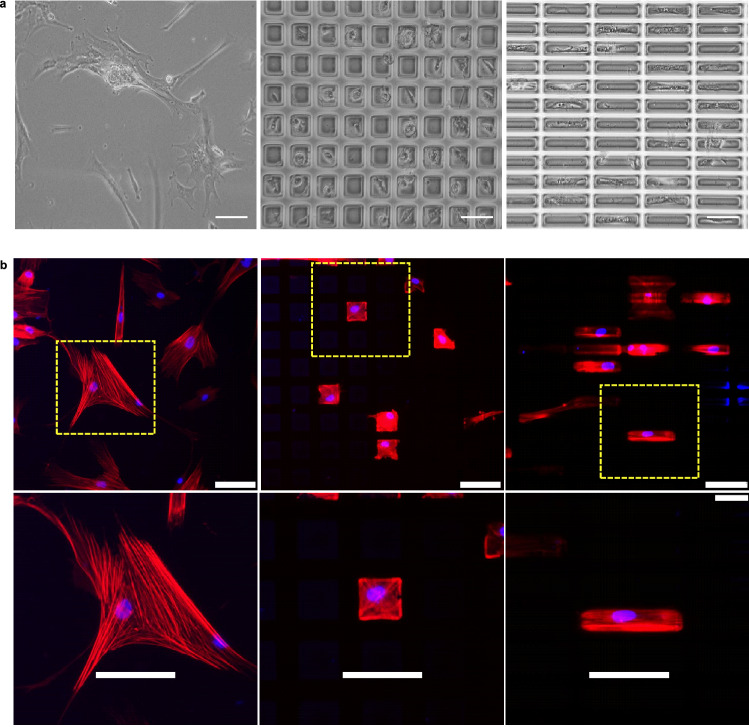
Structure of WJ-MSCs cultured on non-patterned collagen film, 50 μm × 50 μm square collagen microislands, and 120 μm × 20 μm rectangular collagen microislands. (**a**) Phase-contrast microscopy images of WJ-MSCs cultured on the various substrates. Scale bar: 100 μm, cell seeding density: 6 × 10^4^ cells/ml. (**b**) Fluorescence microscopy images of WJ-MSCs cultured on the various substrates. Nucleus (blue) and F-actin (red) in cells were stained by DAPI and phalloidin-conjugated fluorochrome, respectively. Scale bar: 100 μm, cell seeding density: 5 × 10^3^ cells/ml, cell culture time: 5 days.

The biocompatibility of collagen microislands was tested by seeding WJ-MSCs on non-patterned collagen film, square collagen microislands, and rectangular collagen microislands for 5 days. WJ-MSCs cultured on a non-patterned collagen film showed morphologies of elongation or spreading in many directions randomly (Fig. 2a), whereas cells cultured on collagen microislands stayed only on the top of the microislands with the cell size and shape matching that of the microislands underneath (Fig. 2a). To examine cell adhesion status, WJ-MSCs were stained by DAPI and phalloidin conjugated fluorochrome to display cell nucleus and F-actin, respectively. The fluorescence microscopy images showed that WJ-MSCs adhered well on non-patterned collagen film, square (50 μm × 50 μm) collagen microislands, and rectangular (120 μm × 20 μm) collagen microislands (Fig. 2b). The spatial distribution of F-actin revealed that the cell morphology conformed to the extent of the microislands, except that a small percentage of cells could stretch over adjacent microislands. For WJ-MSCs cultured on the non-patterned collagen film F-actin extended in random directions (Fig. 2b). In contrast, for WJ-MSCs cultured on the square microislands F-actin consistently extended from the center to the apexes and also along the edges (Fig. 2b), while for WJ-MSCs cultured on the rectangular collagen microislands F-actin extended along the major axis of the microislands (Fig. 2b). In addition, the positions of the cell nuclei revealed by DAPI staining indicated that 90% of the microislands occupied by cells had only one cell (Fig. 2b). Furthermore, the nuclei of WJ-MSCs cultured on the rectangular microislands became elongated in the direction of cell stretching and F-actin orientation with an aspect ratio of 2.11 ± 0.41 (Fig. 2b), while both the nuclei of WJ-MSCs cultured on the non-patterned collagen film and the square collagen microislands were approximately round with aspect ratios of 1.30 ± 0.15 and 1.26 ± 0.13 respectively (Fig. 2b).

### Optimization of extrinsic photobiomodulation

**Figure 3:**
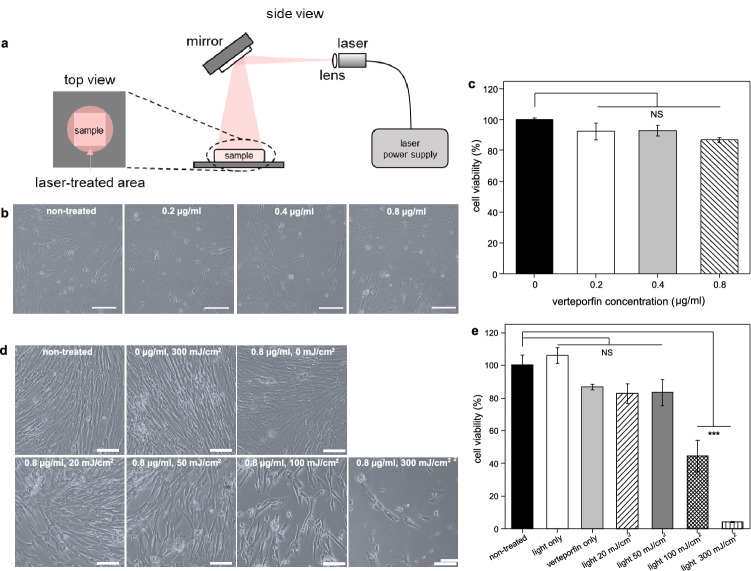
Optimization of verteporfin concentration and light fluence of extrinsic photobiomodulation (EPM). (**a**) Setup of EPM. The light source was a diode laser with a wavelength of 690 nm. The light beam was diverged by using a lens and then reflected downward to the treatment area with a diameter of 8 cm and an intensity (power density) of 5 mW/cm^2^. Samples were placed in the treatment area and the accumulated light fluence (light dose) was controlled by varying the duration of light irradiation. (**b**) WJ-MSC morphology and density observed by phase-contrast microscopy after cultured for 5 days in various concentrations of verteporfin. (**c**) WJ-MSC viability examined by MTT assay after cultured for 5 days in various concentrations of verteporfin. (**d**) WJ-MSC morphology and density observed by phase-contrast microscopy at 5 days after treated with EPM of various conditions: non-treated; treated with light of 300 mJ/cm^2^ fluence without verteporfin (light only); treated with 0.8 μg/ml verteporfin and light of 0, 20, 50, 100, and 300 mJ/cm^2^ fluence, respectively. (**e**) WJ-MSC viability examined by MTT assay at 5 days after treated with EPM of various conditions. Scale bar: 100 μm. Cell seeding density: 2 × 10^4^ cells/ml. Data is expressed by mean ± SD (N = 3), NS: no significant difference, *p value < 0.05; **p value < 0.01; ***p value < 0.001.

To examine the cytotoxicity of extrinsic photobiomodulation (EPM) on WJ-MSCs and thereby determine the suitable condition of EPM, WJ-MSCs were observed with phase-contrast microscopy and the viability was measured with MTT assays after subjected to verteporfin of various concentrations without light irradiation and also light irradiation of various fluences (Fig. 3a) with a chosen verteporfin concentration. At 5 days after treated with verteporfin of 0, 0.2, 0.4, and 0.8 μg/ml concentrations respectively without light irradiation, WJ-MSCs in all groups (non-treated and verteporfin-treated) exhibited elongated, fibroblast-like cell morphology and similar cell density (about 70% confluency) (Fig. 3b), showing that the effect of verteporfin on WJ-MSC morphology and density were insignificant with these concentrations. Quantitatively, the viability of non-treated WJ-MSCs was determined with MTT assay to be 100 ± 1.3% (Fig. 3c), while with verteporfin of 0.2, 0.4, 0.8 μg/ml concentrations the cell viabilities were 92.5 ± 5.5%, 92.9 ± 3.4%, and 86.7 ± 1.7%, respectively, but the differences from the non-treated group were not statistically significant. Both results indicated that the cytotoxicity of verteporfin to WJ-MSCs was negligible for a concentration of 0.8 μg/ml or lower. Therefore, the verteporfin concentration of 0.8 μg/ml was chosen for the subsequent experiments in this study so that the light irradiation time required for effective EPM could be minimized.

Subsequently, the dependence of viability of WJ-MSCs on the light fluence of EPM was studied at a fixed verteporfin concentration of 0.8 μg/ml. A control group without treatment (no verteporfin and light irradiation) and one treated with light irradiation of 300 mJ/cm^2^ fluence but without verteprofin were also prepared for comparison (Fig. 3d, e). After 5 days of incubation, WJ-MSCs in the control groups (non-treated and light only) exhibited elongated, fibroblast-like morphology and occupied the entire substrate (Fig. 3d). With the addition of verteporfin and light irradiation of 20–50 mJ/cm^2^, the cell densities were similar to that of WJ-MSCs cultured in verteporfin without light irradiation (Fig. 3d). However, when the light fluence was increased to 100 mJ/cm^2^ or higher, the number of cells remaining attaching dropped drastically (Fig. 3d). The MTT assay revealed that WJ-MSCs treated by EPM with 20–50 mJ/cm^2^ light fluence did not experience a significant decrease in cell viability (Fig. 3e). In contrast, the viability of WJ-MSCs treated by EPM with 100 mJ/cm^2^ and 300 mJ/cm^2^ light fluences reduced to 40% and 5%, respectively (Fig. 3e). Therefore, a verteporfin concentration of 0.8 μg/ml and a light fluence of 50 mJ/cm^2^ were adopted for the application of EPM in inducing WJ-MSC differentiation so as to maximize the effect while avoiding damaging the cells.

### Optimization of size and shape of collagen microislands

**Figure 4:**
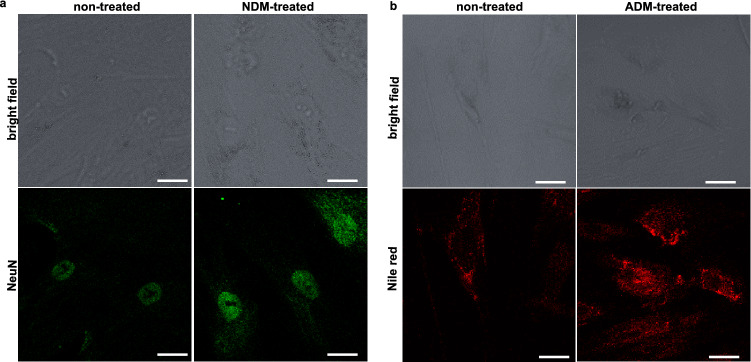
Bright field optical microscopy images and confocal microscopy images of WJ-MSCs cultured with or without inducing chemical factors. (**a**) Neurogenic differentiation, stained by anti-NeuN antibody followed by secondary antibody conjugated fluorochrome (**b**) Adipogenic differentiation, stained by Nile red. Scale bar: 25 μm, cell seeding density: 6 × 10^4^ cells/ml, cell culture time: 10 days for neurogenic differentiation and 5 days for adipogenic differentiation.

For cell differentiation analysis, flow cytometry based on immunostaining of NeuN, a biomarker of mature neural cells^42,43^, and Nile red staining of lipid droplets, a biomarker of adipocytes, was used to quantify neurogenic differentiation rate and adipogenic differentiation rate respectively. A cell was identified as a neural cell/adipocyte if the fluorescence intensity exceeded the threshold, which was selected to include 99% of background measurements. To verify the reliability of the two staining techniques, WJ-MSCs were incubated with and without neurogenic differetiation medium and adipogenic differentiation medium, stained, and measured with confocal microscopy (Fig. 4). The staining of NeuN indicated that NeuN was mainly concentrated in cell nuclei (Fig. 4a) and the staining with Nile red revealed that the lipid was contained in vacuoles in cytoplasm (Fig. 4b), both consistent with their known spatial locations^44,45^. Moreover, the elevated expression of NeuN in WJ-MSCs incubated in neurogenic differentiation medium (Fig. 4a) and that of lipid droplets in WJ-MSCs incubated in adipogenic differentiation medium (Fig. 4b) relative to those in maintenance medium confirmed the observations with flow cytometry.

**Figure 5:**
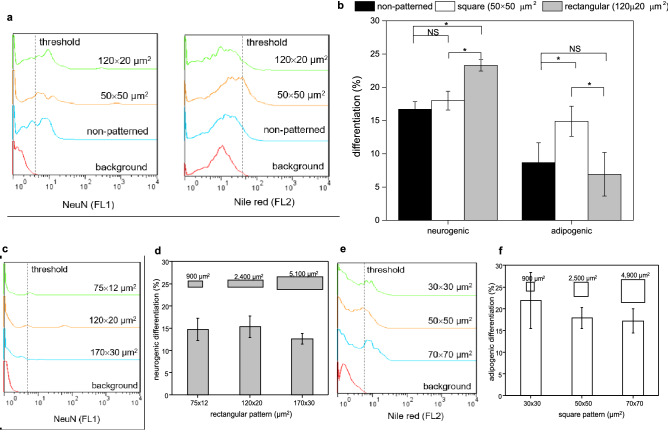
Percentage of WJ-MSCs differentiating into specific lineages induced by corresponding chemical factors (RA cocktail for neurogenic differentiation and Stempro for adipogenic differentiation) and cultured on collagen microislands of various dimensions. (**a**) Representative fluorescent intensity histograms of WJ-MSCs cultured on non-patterned collagen film and collagen microislands of various aspect ratios (AR = 1, 6) with similar areas (2400–2500 μm^2^). (**b**) Neurogenic differentiation rate and adipogenic differentiation rate of WJ-MSCs cultured with the conditions of (**a**). (**c**) Representative fluorescent intensity histograms of WJ-MSCs cultured in RA cocktail on collagen microislands of various areas with roughly the same aspect ratio of 6 (AR = 5.67–6.25). (**d**) Neurogenic differentiation rate of WJ-MSCs cultured with the conditions of (**c**). (**e**) Representative fluorescent intensity histograms of WJ-MSCs cultured in Stempro on collagen microislands of various areas with the same aspect ratio of 1. (**f**) Adipogenic differentiation rate of WJ-MSCs cultured with the conditions of (**e**). Cell seeding density: 6 × 10^4^ cells/ml. Cell culture time: 10 days for neurogenic differentiation and 5 days for adipogenic differentiation. Data is expressed by mean ± SD (N = 4), NS: no significant difference, *p value < 0.05; **p value < 0.01; ***p value < 0.001. The background signal was taken from routinely cultured WJ-MSCs stained with anti-NeuN antibody conjugated fluorochrome or Nile red and then measured with flow cytometry together with the experimental groups.

First, experiments were conducted to determine the optimal aspect ratio and area of collagen microislands that rendered the maximal effect of facilitating WJ-MSC differentiation toward neurogenic and adipogenic differentiations respectively. Comparing the differentiation rates of WJ-MSCs cultured in RA cocktail (neurogenic differentiation medium) on collagen film and collagen microislands of various aspect ratios with roughly the same area (~ 2500 μm^2^), it was found that rectangular microislands with an aspect ratio of 6 could promote neurogenic differentiation more than non-patterned collagen film and square collagen microislands (Fig. 5a, b). In contrast, for WJ-MSCs cultured in Stempro (adipogenic differentiation medium) square microislands displayed a higher efficacy in raising adipogenic differentiation rate than non-patterned collagen film and rectangular collagen microislands (Fig. 5a, b). It could thus be concluded that WJ-MSCs cultured on rectangular microislands preferred neurogenic differentiation while those cultured on square microislands favored adipogenic differentiation.

Next, the dependence of WJ-MSC differentiation rate on the area of collagen microislands was studied with a fixed aspect ratio of 6 (rectangular) for neurogenic differentiation (Fig. 5c, d) and 1 (square) for adipogenic differentiation (Fig. 5e, f). The results showed that the variation of rectangular microisland area from 900 μm^2^ to 5100 μm^2^ had no statistically significant effect on neurogenic differentiation rate (Fig. 5c, d). Similarly, it was also seen that the variation of square microisland area did not influence adipogenic differentiation rate significantly (Fig. 5e, f). These observations suggested that the geometric shape of cell adhesion such as the aspect ratio of rectangle played a significant role in the regulation of WJ-MSC differentiation, while the area seemed to be a minor factor in directing the evolution of the cells. Nevertheless, because the average differentiation rate was higher with 120 μm × 20 μm microislands for neurogenic differentiation and with 30 μm × 30 μm microislands for adipogenic differentiation, the two were chosen for the subsequent experiments that combined collagen microislands with EPM.

### Induction of neurogenic differentiation

**Figure 6:**
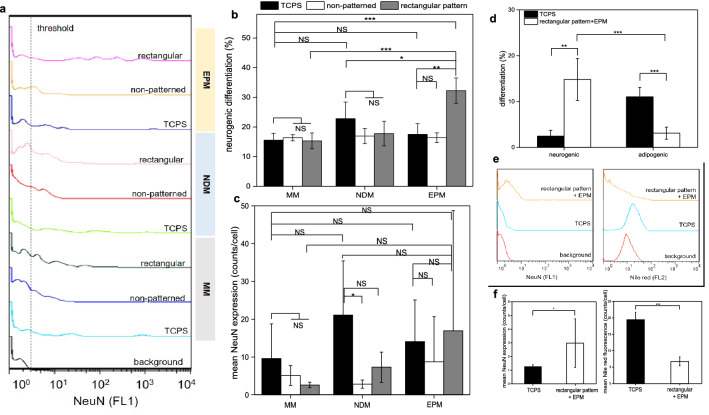
Neurogenic differentiation of WJ-MSCs cultured on TCPS, collagen film, and 120 μm × 20 μm rectangular collagen microislands respectively in maintenance medium (MM), neurogenic differentiation medium (NDM), or subjected to extrinsic photobiomodulation (EPM). (**a**) Representative fluorescent intensity histograms of WJ-MSCs cultured with the various conditions. (**b**) Neurogenic differentiation rate of WJ-MSCs cultured with the various conditions. (**c**) Mean fluorescence intensity of NeuN probe in WJ-MSCs cultured with the various conditions. (**d**) Neurogenic differentiation rate and adipogenic differentiation rate of WJ-MSCs cultured on rectangular microislands with EPM treatment. (**e**) Representative fluorescent intensity histograms of (**d**). (**f**) Mean fluorescence intensity of (**d**). Cell culture time: 10 days. Data is expressed by mean ± SD (N = 4), NS: no significant difference; *p value < 0.05; **p value < 0.01, ***p value < 0.001.

To test the hypothesis that EPM could induce cell differentiation and collagen microislands of designated geometry could specify the direction (outcome) of differentiation, first, WJ-MSCs cultured on tissue culture polystyrene (TCPS), collagen film, and 120 μm × 20 μm rectangular collagen microislands were subjected to EPM in the absence of chemical inducing factors (RA cocktail) and the neurogenic differentiation rates were measured and compared to those with and without chemical factors in the absence of EPM (Fig. 6a, b). It was found that the highest neurogenic differentiation rate was achieved by applying EPM in conjunction with the rectangular collagen microislands (~ 32%), and it was significantly higher than that cultured on TCPS in neurogenic differentiation medium. This revealed that the combination of EPM and the rectangular collagen microislands could render a higher efficacy in inducing neurogenic differentiation than the inducing chemical factors. The fact that there was no significant effect when using just EPM or the rectangular collagen microislands indicated that the two worked synergistically to induce neurogenic differentiation. The mean fluorescence intensity per cell (Fig. 6c) showed a trend similar to the neurogenic differentiation rate (Fig. 6b), although the standard deviations were substantially larger due to the huge variation in the NeuN expression level across many orders of magnitude (Fig. 6a).

Since it has been demonstrated that the geometric cue could affect WJ-MSC differentiation significantly when cultured in differentiation medium (Fig. 5), an experiment was then conducted to test if the direction of differentiation could be controlled by the geometric cue from the collagen microislands in this modality. It was found that WJ-MSCs cultured on the rectangular microislands with EPM treatment could promote neurogenic differentiation (~ 15%) much more than adipogenic differentiation (~ 3%) (Fig. 6d). The latter was even lower than the control group cultured on TCPS with just maintenance medium, indicating that the geometric cue could even suppress the spontaneous differentiation in the unmatched directions (Fig. 6d, e). The mean fluorescence intensity per cell (Fig. 6f) showed a trend similar to the differentiation rate (Fig. 6d). These results verified that EPM played the role of inducing WJ-MSC differentiation while the geometric cue from collagen microislands determined the direction of differentiation.

**Figure 7:**
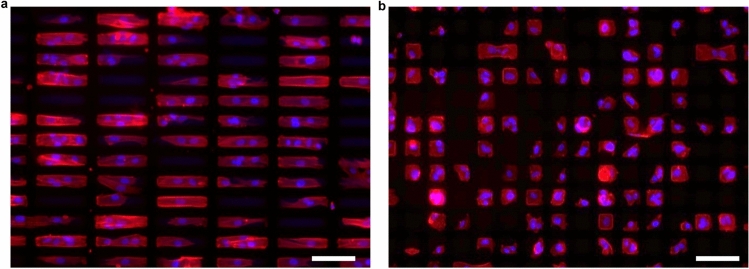
Confocal microscopy images of WJ-MSCs cultured on collagen microislands of different dimensions. (**a**) 120 μm × 20 μm, (**b**) 30 μm × 30 μm. Nuclei and F-actin in cells were stained by DAPI and phalloidin-conjugated fluorochrome, respectively. Scale bar: 100 μm, cell seeding density: 6 × 10^4^ cells/ml, cell culture time: 5 days.

It was noted that the neurogenic differentiation rate of WJ-MSCs cultured on the rectangular microislands with EPM shown in Fig. 6d was half of that shown in Fig. 6b. It could be attributed to the substantially higher number of cells in the latter compared to that in the former such that a significant percentage of collagen microislands were occupied by two or more cells (Fig. 7), which led to limited elongation of the cells and thus diminished the effect of the geometric cue, as can be seen from the round nuclei in such cases in contrast to the elongated nuclei shown in Fig. 2b. In general, for the method of EPM in conjunction with collagen microislands a higher cell density would decrease the differentiation rate. This is because when the cell density gets higher, the percentage (probability) of collagen microislands occupied by two or more cells would increase, leading to a cell morphology that does not conform to the optimal microisland topography for the target differentiation outcome and thus diminished the effect of the geometric cue provided by the microislands for the cells in that situation. Taking the case of neurogenic differentiation for example, an aspect ratio of 6 was found to render the highest neurogenic differentiation rate compared to those with smaller aspect ratios. When there were two cells occupying the same microisland, each of them could only occupy half of the microisland and thus the actual aspect ratio of the morphology of each cell was only about 3 instead of 6, so the neurogenic differentiation rate would be decreased significantly. The higher cell density of the experiment of Fig. 6d could be ascribed to the use of WJ-MSCs with a substantially higher passage number, which were observed to display stronger adhesion on a substrate (as revealed by a longer time taken for the cells to detach from TCPS upon trypsin treatment during routine cell passages) compared to those with a lower passage number and thus could be better retained on the collagen microislands. Therefore, a lower cell seeding density should be used when the cell passage number is higher in order to render a higher differentiation rate by minimizing the number of microislands occupied by more than one cell.

### Induction of adipogenic differentiation

**Figure 8:**
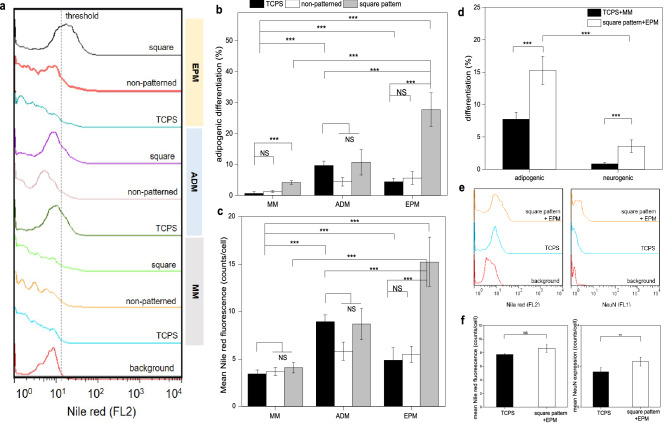
Adipogenic differentiation of WJ-MSCs cultured on TCPS, collagen film, and 30 μm × 30 μm square collagen microislands respectively in maintenance medium (MM), adipogenic differentiation medium (ADM), or subjected to extrinsic photobiomodulation (EPM). (**a**) Representative fluorescent intensity histograms of WJ-MSCs cultured with the various conditions. (**b**) Adipogenic differentiation rate of WJ-MSCs cultured with the various conditions. (**c**) Mean fluorescence intensity of Nile red in WJ-MSCs cultured with the various conditions. (**d**) Adipogenic differentiation rate and neurogenic differentiation rate of WJ-MSCs cultured on square microislands with EPM treatment. (**e**) Representative fluorescent intensity histograms of (**d**). (**f**) Mean fluorescence intensity of **d**. Cell culture time: 5 days. Data is expressed by mean ± SD (N = 4), NS: no significant difference, *p value < 0.05; **p value < 0.01; ***p value < 0.001.

To test the generality of this modality of EPM in conjuction with collagen microislands of designated geometric cue for different differentiation outcomes, WJ-MSCs cultured on TCPS, collagen film, and 30 μm × 30 μm square collagen microislands were subjected to EPM in the absence of inducing chemical factors (Stempro) and the adipogenic differentiation rates were measured and compared to those with and without chemical factors in the absence of EPM (Fig. 8a, b). It was found that the highest adipogenic differentiation rate was achieved by applying EPM in conjunction with the square collagen microislands (~ 28%), and it was significantly higher than that cultured on TCPS in adipogenic differentiation medium. This revealed that the combination of EPM and the square collagen microislands could render a higher efficacy in inducing adipogenic differentiation than the inducing chemical factors. Although the effect of using just EPM or just the square collagen microislands was also statistically significant, the effect of the combination was much stronger than the sum of the two working alone, indicating that the two factors could work synergistically to induce adipogenic differentiation more prominently. The mean fluorescence intensity per cell (Fig. 8c) showed a trend similar to the adipogenic differentiation rate (Fig. 8b).

Likewise, an experiment was then conducted to test if the direction of differentiation could be controlled by the geometric cue from the collagen microislands in this modality. It was found that WJ-MSCs cultured on the square microislands with EPM treatment could promote adipogenic differentiation (~ 15%) much more than neurogenic differentiation (~ 4%) (Fig. 8d, e). The mean fluorescence intensity per cell (Fig. 8f) showed a trend similar to the differentiation rate (Fig. 8d). These results again verified that EPM played the role of inducing WJ-MSC differentiation while the geometric cue from collagen microislands determined the direction of differentiation. Note that in this case the neurogenic differentiation rate was higher than that of the control group cultured on TCPS with just maintenance medium, this could be ascribed to the elevated expression of NeuN in adipogenically differentiated human mesenchymal stem cells as reported previously by other groups^43^.

To obtain some preliminary information on the underlying machinery of this method, following the insights gained from the past work on inducing stem cell differentiation by chemical factors^46^, measurements of cell plasma membrane potential on day 0 and day 5 for the groups of MM, ADM, and EPM in combination with square microislands were conducted (Supplementary Fig. S2). Consistent with the past work^46^, the ADM group exhibited a hyperpolarization after 5 days with respect to the membrane potential of WJ-MSCs (day 0). More importantly, the EPM + square microisland group showed an even more dramatic hyperpolarization (a larger drop in DiSBAC_2_(3) fluorescence intensity), in agreement with the higher adipogenic differentiation rate shown in Fig. 8. Since the past work has proven by pharmacological interference that the change of plasma membrane potential is a key intermediate in the signaling cascade that drives mesenchymal stem cell differentiation, this result indicated that the underlying machinery of inducing WJ-MSC differentiation by EPM in combination with collagen microislands was also mediated by cell membrane potential.

## Discussion

It has been well recognized that the size and geometric shape of cell adhesion dictated by substrate pattern can facilitate or polarize stem cell differentiation in favor of a specific outcome^17–19,21^. In this work, with collagen microislands fabricated by photolithography and collagen deposition (Fig. 1), WJ-MSCs were made to adhere onto the microislands with their size and shape conforming to that of the microislands (Fig. 2). Under the influence of differentiation-inducing chemical factors, the rectangular microislands with an aspect ratio of 6 facilitated neurogenic differentiation more than non-patterned substrate and square microislands, while square microislands promoted adipogenic differentiation better than non-patterned substrate and rectangular microislands (Fig. 5). It was also observed that the differentiation rate seemed to be dependent on the area of the microisland as reported by other groups, although the effect was not statistically significant in our case (Fig. 5). It can be concluded that collagen microislands of different areas and aspect ratios favor different differentiation outcomes, with the aspect ratio playing a more prominent role. These results are consistent with the findings of previous reports that the lineage commitment of stem cells can be directed by geometric cues^12,16,17,21^. The mechanism could be attributed to the control of F-actin distribution by cell adhesion morphology through the interaction of F-actin, integrin, and extracellular matrix, which in turn leads to change in chromosome territory distribution and thus change in gene expression. This explanation is supported by the observed correspondence between cell adhesion morphology, F-actin distribution, and the shape of cell nucleus (Fig. 2b). High-resolution imaging of chromosome territory distribution and its map of activation would be helpful to provide the final link to the change of cell phenotype.

The most important finding of this work is that the application of EPM in conjunction with collagen microislands can induce WJ-MSC differentiation in the direction specified by the microisland pattern and the differentiation rate can surpass that obtained with differentiation-inducing chemical factors. For neurogenic differentiation the combination of EPM and rectangular microislands exhibited a differentiation rate that was 2 and 1.5 times that achieved without and with using neurogenic differentiation medium respectively (Fig. 6). Likewise, for adipogenic differentiation the combination of EPM and square microislands rendered a differentiation rate that was 30 and 3 times that achieved without and with using adipogenic differentiation medium respectively (Fig. 8). Moreover, for adipogenic differentiation both EPM and optimal microislands alone could raise the differentiation rate and the combination worked synergistically to achieve a higher rate in a multiplicative way. However, for neurogenic differentiation both EPM and optimal microislands had insignificant effects in increasing differentiation rate, but the combination was able to double the differentiation rate. All these results seem to indicate that EPM could trigger WJ-MSC differentiation while collagen microislands could determine the direction of differentiation with a unique optimal island pattern designated for each cell phenotype. The latter was also confirmed by the specificity tests (Figs. 6d, 8d). This method of inducing stem cell differentiation not only could achieve a higher efficacy than using chemical factors, but also provides a more universal way of controlling stem cell differentiation. In contrast to the induction of stem cell differentiation using chemical factors, which requires finding a specific recipe for each cell phenotype, this modality has the potential to be applicable to various cell phenotypes simply by using collagen microislands of corresponding patterns. Application of this method to other differentiation targets would further corroborate its generality. Induction of direct lineage reprogramming of somatic cells with this method seems possible and should be explored in order to expand its practical application in tissue engineering.

The ability of EPM to initiate WJ-MSC differentiation may be ascribed to induction/alteration of oscillation/spiking of intracellular Ca^2+^ concentration. It has been reported that stem cells exhibit a change in Ca^2+^ oscillation upon the induction of differentiation with chemical factors and the manipulation of Ca^2+^ oscillation with electrical stimulation can substitute for the chemical factors to drive stem cell differentiation^47^. It has also been shown that intrinsic photobiomodulation can induce an oscillation in Ca^2+^ concentration^48–50^ and has the ability to facilitate stem cell differentiation^25–30^. In addition, it was found that the ability of substrate rigidity to regulate stem cell differentiation is mediated by Ca^2+^ oscillation^51^. Therefore, it seems reasonable to assume that the EPM used here was able to trigger/alter Ca^2+^ oscillation and thus initiate the differentiation process. This hypothesis is supported by a previous report showing that the photosensitizer verteporfin used here binds to endoplasmic reticulum, which is the major intracellular Ca^2+^ storage, and can dump Ca^2+^ into the cytoplasm upon light irradiation^52^. The capability of Ca^2+^ oscillation/modulation in driving stem cell differentiation could be attributed to the universality of Ca^2+^ as an intracellular messenger. The calcium signaling toolkit possesses many components that can be concerted to create a wide range of spatial and temporal signals^53,54^, which may dynamically coordinate the biochemical processes involved in cell differentiation. In this work, it was found that EPM in conjunction with collagen microisland led to a larger hyperpolarization of WJ-MSCs than that achieved by ADM after 5 days (Supplementary Fig. S2), which correlated well with the higher adipogenic differentiation rate attained by the former. It has been shown that a change of plasma membrane potential of mesenchymal stem cell resulted in a change of Ca^2+^ oscillation pattern^55^ and that the two could affect each other in a feedback-controlled manner^56^. Further investigation is required to elucidate the details of the intracellular signaling dynamics driven by Ca^2+^ oscillation in response to EPM and the interplay between Ca^2+^ oscillation and cell adhesion morphology that achieves a synergistic effect in activating and controlling stem cell differentiation.

## Methods

### Stem cell culture

Human umbilical cord Wharton’s Jelly mesenchymal stem cells (WJ-MSCs) were purchased from Bioresource Collection and Research Center (BCRC), Taiwan. WJ-MSCs were cultured in maintenance medium (MM) consisting of Eagle’s minimum essential medium (EMEM, HyClone) supplemented with 20% fetal bovine serum (FBS, SAFC), 4 ng/ml human fibroblast growth factor-basic (bFGF, Peprotech) and 1% antibiotic (Penicillin-Streptomycin solution, Sigma) in tissue culture polystyrene (TCPS) flask (Biofil). Cell culturing was conducted in a humidified atmosphere of 5% CO_2_ at 37 °C and sub-cultured 2–3 times every week. When >80% confluency was reached, cells were detached by using 0.05% trypsin (Biological Industries), centrifuged in order to remove the residual trypsin solution, and then transferred to new TCPS flasks.

### Induction of WJ-MSC differentiation with chemical factors

For induction of differentiation by chemical factors, WJ-MSCs with appropriate cell densities were seeded on various substrates and cultured in maintenance medium for 24 h to allow cell attachment and then the medium was replaced by that containing inducing chemical factors. For neurogenic differentiation, WJ-MSCs were cultured in neurogenic differentiation medium (NDM) consisting of 5 μM retinoic acid (RA, Sigma), 10 ng/ml bFGF, 0.1 mM ascorbic acid (J.Y.Baker), 0.1 mM 3-isobutyl-1-methylxanthine (IBMX, Alfa Aesar)^57^ in EMEM supplemented with 20% FBS and 1% antibiotic during the whole period of 10 days. The NDM was replaced with fresh NDM every 2–3 days. For adipogenic differentiation, WJ-MSCs were first cultured with commercial adipogenic differentiation medium (ADM) (Stempro Adipogenic Differentiation Kit, Gibco) for 3 days and then with maintenance medium for 2 days. The changes of medium were done by aspiration and replenishing without disrupting the attachment of cells.

### Preparation of collagen microislands and collagen film

Glass substrates cut from glass slides (Green Cross) in dimensions of 0.9 cm × 0.9 cm were cleaned by sonication using Glass-Klenz (Steris), ethanol, acetone, and deionized water sequentially. Each sonication step took 15 min. The pristine glass substrates were then exposed to oxygen plasma with 20 sccm flow rate, 100 mTorr pressure, and 40 W power for 1 min and then treated with 1% 3-(trimethoxysilyl)propyl methacrylate (TMA, Alfar Aesar) in toluene for 1 h, followed by washing twice with toluene and deionized water. Afterwards, 15 μl of the mixture of 25 v/v% poly(ethylene glycol) diacrylate (PEGDA, Sigma) and 1 v/v% of 2-hydroxyl-2-methylpropiophenone (HOMPP, Sigma) was dropped on each silanized glass substrate. A Cr/fused silica photomask containing 12 regions of rectangular patterns with different dimensions was placed on top of the PEGDA-HOMPP-covered glass substrates, with the region of choice superimposed on the substrates and the photomask suspended by a blank glass substrate and a spacer of 6 μm thickness at each corner. The region of choice was then exposed to UVA light of 365 nm wavelength for 25 s from above with the distance between the photomask and the UVA lamp set at 5 cm. After UVA exposure, the samples and photomask were immersed in deionized water for 10 min to remove uncross-linked PEGDA and detach the samples from the photomask. PEGDA microislands were formed on the substrates. After washing with deionized water, the samples were stored in hydrated condition until use to avoid shrinking and spallation of the microislands upon drying. A collagen solution of 6 mg/ml collagen concentration with pH = 2 (Sigma) was diluted to a concentration of 120 μg/ml and then stored at 4 °C, referred to as collagen stock solution. A buffer solution was prepared with 60 mM KH_2_PO_4_, 60 mM Na_2_HPO_4_, and 250 mM KCl, referred to as PK buffer. The collagen stock solution and the PK buffer were mixed in a ratio of 1:5 to produce a collagen working solution with 20 μg/ml collagen and pH = 8. To prepare collagen microislands, 500 μl of collagen working solution was dropped onto the surface of a PEGDA microisland substrate, and the sample was then incubated in a cell incubator (37 °C and humidified) for 2 h. After washing by deionized water, a collagen microisland substrate was obtained. For making collagen film the same process was applied except that the photomask was replaced by a blank (transparent) glass so that the entire substrate was covered with cured PEGDA.

For all cell differentiation experiments using collagen film or collagen microisland substrates, the substrate was first placed in a well (15.6 mm diameter) of a 24-well TCPS plate and then WJ-MSCs with appropriate cell densities were seeded and cultured in maintenance medium for 24 h to allow cell attachment. After that, the cell-loaded substrate was transferred to a well (1 cm × 1 cm) of a 8-well plate to start the differentiation period with or without applying EPM or inducing chemical factors. This was to ensure that the cells that were not loaded onto the substrate did not interfere with the measurement of differentiation rates using flow cytometry.

### Extrinsic photobiomodulation

For applying extrinsic photobiomodulation, WJ-MSCs were cultured on various substrates in maintenance medium containing 0.8 μg/ml verteporfin (Sigma) for 1 h and then irradiated with a laser beam of 690 nm wavelength for an accumulated light fluence (dose) of 50 mJ/cm^2^ (light intensity: 5 mW/cm^2^, duration: 10 s). Afterwards, the sample was incubated at 37 °C for 1 h and then the medium containing verteporfin was removed and replaced by maintenance medium. For all cell differentiation experiments, to render a higher efficacy, extrinsic photobiomodulation was applied every 2–3 days whenever the medium was replaced.

### Cell viability measurement

MTT assays were used for measurement of cell viability. The medium of the cell culture was removed and replaced by 500 μl of 5 mg/ml 3-(4,5-dimethylthiazol-2-yl)-2,5-diphenyltetrazolium bromide (MTT) in phosphate-buffered saline (PBS). After incubated for 4 h in the cell incubator, the MTT solution was removed and dimethyl sulfoxide (DMSO) was added to solubilize the formazan crystallites formed in the cytoplasms of living cells. Then, 200 μl of the solution was transferred to a 96-well microtiter plate and the optical density (OD) was measured at 560 nm wavelength using a spectrophotometer (Biorad). The cell viability was calculated by dividing the OD of the experimental group by that of the control group which was not treated by verteporfin or light irradiation.

### Collagen imaging

The spatial distribution of collagen on a microisland substrate was imaged by using collagen immunostaining. First, the sample was fixed in 3.7% formaldehyde for 10 min, followed by washing with PBS three times. Next, 0.5 w/v% bovine serum albumin (BSA, Sigma) in PBS was added to block non-specific binding sites and then removed after 1 h. Afterwards, rabbit anti-collagen I antibody (Abcam) was added to the sample and then the sample was incubated overnight at 4 °C. In the following day, the medium containing first antibody was removed and the sample was washed three times with PBS. Finally, the sample was incubated with goat anti-rabbit antibody conjugated Alexa Fluor 594 (Abcam) for 1 h at room temperature. After removing the medium, the sample was washed three time using PBS and then observed in PBS with a confocal microscope (Leica SP8). Both primary and secondary antibodies were diluted in 1:500 ratio. The entire process was executed in a light-tight environment.

### Cell imaging

The distribution of DNA and F-actin in cells were stained by 4′,6-diamidino-2-phenylindole (DAPI, Sigma) and phalloidin conjugated tetramethylrhodamine B isothiocyanate (Sigma), respectively. WJ-MSCs on various substrates were fixed with 3.7% formaldehyde for 10 min and then washed with PBS three times. Afterwards, the sample was permeabilized with 0.1% Triton X-100 (Sigma) for 10 min, followed by washing with PBS three times. Next, 0.5 w/v% BSA in PBS was added and the sample was incubated overnight at 4 °C. In the following day, the sample was incubated with DAPI (1:500 dilution) and phalloidin-conjugated fluorochrome (1:500 dilution) for 2 h at room temperature in the dark and then observed with a fluorescence microscope (Olympus IX73). The dimensions of the cell nuclei revealed by DAPI staining was measured by using ImageJ software, and the aspect ratio of a cell nucleus was defined as the ratio of major axis length (the largest width) to minor axis length (the smallest width) of an elliptically shaped nucleus. The mean ± SD was calculated from the aspect ratios of the nuclei of 50 cells.

### Characterization of cell differentiation

Quantitative analyses of cell differentiation were conducted by using flow cytometry. Cells on various substrates were detached by trypsinization and collected by centrifugation. For characterization of neurogenic differentiation, cells were then fixed by 3.7% formaldehyde and permeabilized by 0.1% Triton X-100. Next, cells were incubated with rabbit anti-NeuN antibody (1:500 dilution, Invitrogen) at room temperature for 1 h and then collected by centrifugation. Afterwards, cells were stained with goat anti-rabbit antibody conjugated with Alexa Flour 488 (Invitrogen) and then measured with a flow cytometer (FACSCalibur, BD) using FL1 channel. For characterization of adipogenic differentiation, cells were incubated with Nile red solution (1:500 dilution, Abcam) at room temperature for 1 h and then measured with the flow cytometer using FL2 channel. Routinely cultured (undifferentiated) WJ-MSCs stained with the same protocols and measured in the same batch were used to determine the background signals for both measurements in order to set a threshold for identifying the cell types. For confocal microscopy measurement the same staining protocols were applied in situ without detaching the cells and then the cells were imaged with a confocal microscope (Leica SP8).

### Measurement of cell membrane potential

To measure the change in cell membrane potential caused by the various treatments, cells on various substrates were washed by Hanks’ balanced salt solution (HBSS, Thermofisher) and then stained with 0.5 μM bis-(1,3-diethylthiobarbituric acid)trimethine oxonol (DiSBAC_2_(3), Thermofisher) for 30 min. Images were taken by confocal microscope (Leica SP8) with an excitation wavelength of 530 nm and an emission collection band of 565–575 nm. ImageJ software was used to specify a region of interest enclosing each individual cell and then obtain the integrated fluorescence signal from each cell. DiSBAC_2_(3) is a potential-sensitive fluorescent dye which can enter depolarized cells where it binds to intracellular proteins or membrane and exhibits enhanced fluorescence. Increased depolarization results in additional influx of the anionic dye and an increase in fluorescence, and, conversely, hyperpolarization is indicated by a decrease in fluorescence.

### Statistical analyses

Statistical analyses were performed by one-way analysis of variance (ANOVA) executed by Origin software. Tukey comparison tests were utilized to quantify the statistical significance of the differences among samples. The degree of statistical significance was indicated in the graphs by asterisk marks: * for p value < 0.05, ** for p value < 0.01, *** for p value < 0.001, NS: no significant difference.

## Supplementary Information

Supplementary Information.
