# Supplementary material for: Control of stem cell differentiation by using extrinsic photobiomodulation in conjunction with cell adhesion pattern
Source: Sci Rep. 2022 Feb 2;12:1812. doi: 10.1038/s41598-022-05888-3 (PMC8811059; doi:10.1038/s41598-022-05888-3)
Supplement: Supplementary file 1 — Supplementary Information. [file 41598_2022_5888_MOESM1_ESM.pdf]

Supplementary Information

Control of stem cell differentiation by using extrinsic photobiomodulation in conjunction with cell adhesion pattern

Saitong Muneekaew, Meng-Jiy Wang, Szu-yuan Chen

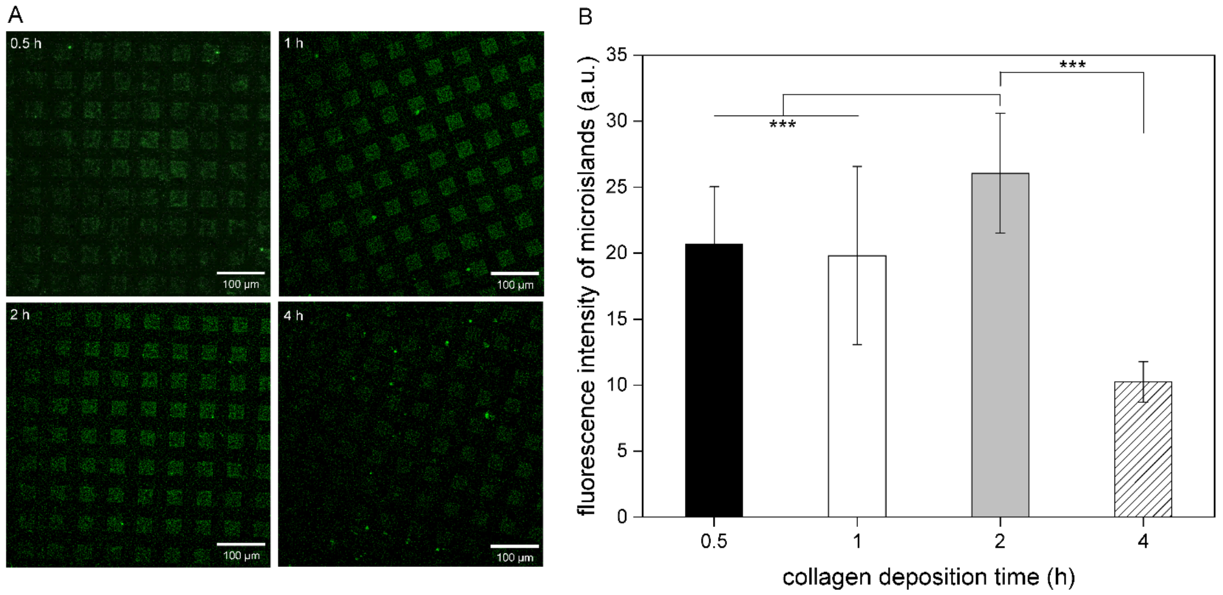

Supplementary Figure S1 Optimization of collagen deposition time. (A) Images of immunostaining of collagen deposited on PEGDA microislands with various deposition times. (B) Fluorescence intensity of microislands as a function of collagen deposition time. Data is expressed by mean ± SD (N=50).

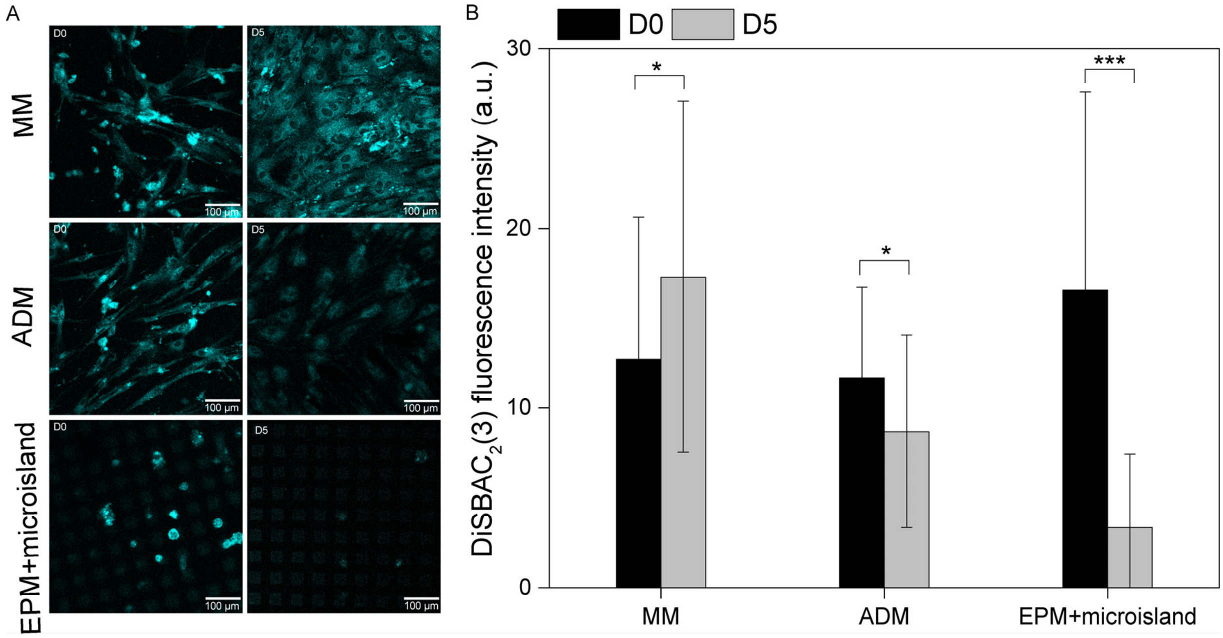

Supplementary Figure S2 Measurement of cell membrane potential by DiSBAC$_2$(3) staining. (A) Images of DiSBAC$_2$(3) staining of WJ-MSCs cultured in various conditions. MM: on glass substrate in maintenance medium; ADM: on glass substrate in adipogenic differentiation medium for 3 days and then in maintenance medium for 2 days; EPM+microislands: on 30 μm × 30 μm square collagen microislands in maintenance medium and treated by extrinsic photobiomodulation (EPM) on day 0 and day 3. D0: taken right after the treatment with differentiation medium or EPM. D5: taken after 5 days. (B) Total DiSBAC$_2$(3) fluorescence intensity of cells retrieved from fluorescence images by using ImageJ software. Data is expressed by mean ± SD (N=50, randomly select 10 cells each from 5 samples).
